# Supplementary material for: Multi‐dataset identification of innovative feature genes and molecular mechanisms in keratoconus
Source: J Cell Mol Med. 2024 Sep 19;28(18):e70079. doi: 10.1111/jcmm.70079 (PMC11412914; doi:10.1111/jcmm.70079)
Supplement: Supplementary file 2 — Material S2. [file JCMM-28-e70079-s002.zip › 7.GSEA analysis/5.RNF217-AS1/KEGG_SYSTEMIC_LUPUS_ERYTHEMATOSUS.html]

Details for gene set KEGG\_SYSTEMIC\_LUPUS\_ERYTHEMATOSUS[GSEA]

|  || Dataset | RNF217-AS1.group.cls#h\_versus\_l |
| Phenotype | group.cls#h\_versus\_l |
| Upregulated in class | l |
| GeneSet | KEGG\_SYSTEMIC\_LUPUS\_ERYTHEMATOSUS |
| Enrichment Score (ES) | -0.51660734 |
| Normalized Enrichment Score (NES) | -1.2572935 |
| Nominal p-value | 0.21774194 |
| FDR q-value | 0.43995816 |
| FWER p-Value | 0.968 |
Table: GSEA Results Summary

  

Fig 1: Enrichment plot: KEGG\_SYSTEMIC\_LUPUS\_ERYTHEMATOSUS      
 Profile of the Running ES Score & Positions of GeneSet Members on the Rank Ordered List

  

| SYMBOL | TITLE | RANK IN GENE LIST | RANK METRIC SCORE | RUNNING ES | CORE ENRICHMENT || 1 | CD86 | na | 733 | 0.481 | 0.0306 | No |
| 2 | ELANE | na | 6064 | 0.181 | -0.1037 | No |
| 3 | C6 | na | 6523 | 0.167 | -0.0989 | No |
| 4 | C8A | na | 7260 | 0.145 | -0.1045 | No |
| 5 | C7 | na | 7568 | 0.138 | -0.0985 | No |
| 6 | C8G | na | 7627 | 0.137 | -0.0855 | No |
| 7 | CD28 | na | 9337 | 0.098 | -0.1242 | No |
| 8 | GRIN2A | na | 9393 | 0.097 | -0.1153 | No |
| 9 | CTSG | na | 9428 | 0.097 | -0.1059 | No |
| 10 | CD80 | na | 10316 | 0.081 | -0.1228 | No |
| 11 | C9 | na | 10508 | 0.078 | -0.1199 | No |
| 12 | FCGR2A | na | 11203 | 0.067 | -0.1327 | No |
| 13 | ACTN4 | na | 11251 | 0.067 | -0.1269 | No |
| 14 | ACTN1 | na | 11587 | 0.061 | -0.1300 | No |
| 15 | HLA-DOA | na | 11639 | 0.061 | -0.1250 | No |
| 16 | SNRPD3 | na | 13265 | 0.039 | -0.1676 | No |
| 17 | ACTN3 | na | 13294 | 0.039 | -0.1643 | No |
| 18 | TNF | na | 13583 | 0.035 | -0.1688 | No |
| 19 | HLA-DMB | na | 13802 | 0.033 | -0.1716 | No |
| 20 | FCGR1A | na | 14177 | 0.029 | -0.1793 | No |
| 21 | C1QC | na | 14192 | 0.028 | -0.1767 | No |
| 22 | HLA-DMA | na | 14233 | 0.028 | -0.1748 | No |
| 23 | C8B | na | 15799 | 0.011 | -0.2188 | No |
| 24 | SSB | na | 16649 | 0.002 | -0.2430 | No |
| 25 | ACTN2 | na | 16708 | 0.002 | -0.2446 | No |
| 26 | C4A | na | 17397 | -0.005 | -0.2638 | No |
| 27 | SNRPD1 | na | 18781 | -0.018 | -0.3017 | No |
| 28 | SNRPB | na | 19007 | -0.020 | -0.3061 | No |
| 29 | HLA-DRB5 | na | 19471 | -0.025 | -0.3167 | No |
| 30 | FCGR2B | na | 21452 | -0.045 | -0.3690 | No |
| 31 | HLA-DRB1 | na | 23407 | -0.067 | -0.4182 | No |
| 32 | CD40 | na | 23601 | -0.070 | -0.4163 | No |
| 33 | HLA-DRB3 | na | 23619 | -0.070 | -0.4093 | No |
| 34 | C4B | na | 23794 | -0.072 | -0.4065 | No |
| 35 | GRIN2B | na | 26448 | -0.115 | -0.4706 | No |
| 36 | HLA-DOB | na | 26792 | -0.122 | -0.4674 | No |
| 37 | C1QA | na | 27255 | -0.131 | -0.4667 | No |
| 38 | TRIM21 | na | 27658 | -0.140 | -0.4632 | No |
| 39 | IL10 | na | 28464 | -0.161 | -0.4692 | No |
| 40 | CD40LG | na | 30110 | -0.210 | -0.4940 | Yes |
| 41 | HLA-DRB4 | na | 30111 | -0.210 | -0.4715 | Yes |
| 42 | HLA-DPA1 | na | 30869 | -0.237 | -0.4679 | Yes |
| 43 | HLA-DQB1 | na | 31211 | -0.250 | -0.4508 | Yes |
| 44 | HLA-DRA | na | 31261 | -0.252 | -0.4251 | Yes |
| 45 | FCGR2C | na | 31803 | -0.279 | -0.4107 | Yes |
| 46 | C1R | na | 32064 | -0.295 | -0.3864 | Yes |
| 47 | HLA-DQA1 | na | 32266 | -0.307 | -0.3592 | Yes |
| 48 | C1QB | na | 32406 | -0.317 | -0.3291 | Yes |
| 49 | C5 | na | 32605 | -0.332 | -0.2992 | Yes |
| 50 | FCGR3A | na | 32899 | -0.353 | -0.2698 | Yes |
| 51 | HLA-DQA2 | na | 33332 | -0.388 | -0.2405 | Yes |
| 52 | IFNG | na | 33359 | -0.391 | -0.1992 | Yes |
| 53 | HLA-DPB1 | na | 33623 | -0.423 | -0.1614 | Yes |
| 54 | C2 | na | 33778 | -0.443 | -0.1182 | Yes |
| 55 | C1S | na | 34217 | -0.532 | -0.0736 | Yes |
| 56 | C3 | na | 34660 | -0.822 | 0.0020 | Yes |
Table: GSEA details [plain text format]

  

Fig 2: KEGG\_SYSTEMIC\_LUPUS\_ERYTHEMATOSUS      
 Blue-Pink O' Gram in the Space of the Analyzed GeneSet

  

Fig 3: KEGG\_SYSTEMIC\_LUPUS\_ERYTHEMATOSUS: Random ES distribution      
 Gene set null distribution of ES for **KEGG\_SYSTEMIC\_LUPUS\_ERYTHEMATOSUS**
